# Identification of an immune subtype predicting survival risk and immune activity in hepatocellular carcinoma

**DOI:** 10.18632/aging.202953

**Published:** 2021-05-03

**Authors:** Feng Zhao, Nuo Xu, Jiali Yang, Bo Li, Jianwei Shi, Yuanyuan Zheng, Longjin Xu

**Affiliations:** 1 Department of Central Laboratory, Huaian Tumor Hospital and Huaian Hospital of Huaian, Huaian 223200, Jiangsu, China; 2 Department of Interventional Radiology, Huaian Tumor Hospital and Huaian Hospital of Huaian, Huaian 223200, Jiangsu, China; 3 Queen Mary University of Nanchang, Nanchang 330031, Jiangxi, China; 4 Department of Ultrasonography, Huaian Tumor Hospital and Huaian Hospital of Huaian, Huaian 223200, Jiangsu, China; 5 Toxicology and Functional Laboratory, Shandong Center for Disease Control and Prevention, Jinan 250014, Shandong, China

**Keywords:** hepatocellular carcinoma, immune subtyping, prognosis, immunotherapy, microenvironment

## Abstract

Immune checkpoint inhibitors (ICI) prolong the survival for advanced/metastatic patients with lung cancer or melanoma; however, for hepatocellular carcinoma (HCC) patients, a durable response has not been reported. Herein, we used a total of 719 HCC patients with public genomic data to determine potential prognostic and immunogenic subtypes. The non-negative matrix factorization (NMF) method was applied to identify the immune classes and potential subtypes. The proportion of tumor infiltration immune cells was estimated using the CIBERSORT algorithm. Gene set enrichment analysis (GSEA) was utilized to calculate the dysregulated pathways. By using NMF analysis for the gene expression profile of the top immune genes, one HCC subtype with better survival (i.e., low-risk subtype) and another with worse survival (i.e., high-risk subtype) were identified in 3 HCC cohorts (all *P* < 0.05). Better immune cell infiltration, increased enrichment of immune signatures, higher expression of checkpoints, and elevated tumor mutation load (TML) were significantly enriched in the low-risk subtype (all *P* < 0.05). Higher mutation rates of immune response genes (e.g., *TP53* and *MUC16*) were also observed in the low-risk subtype (both *P* < 0.05). Discovery of the HCC low-risk subtype might provide clues for HCC prognosis and immunotherapy prediction.

## INTRODUCTION

Hepatocellular carcinoma (HCC) is a common digestive cancer and the second leading cause of cancer-related death around the world. The number of deaths of HCC patients gradually increases every year, indicating its high lethality [1, 2]. This tumor always emerges in the background of chronic liver disease (e.g., cirrhosis) and is correlated with several well-known factors, such as hepatitis B virus (HBV), hepatitis C virus (HCV), alcohol consumption, diabetes mellitus, and metabolic syndrome [2]. In recent years, novel and fast-growing medical technologies have illuminated the molecular mechanisms underlying the occurrence and development of HCC; however, the current clinical therapeutic methods are limited [2, 3]. Only a subset of HCC patients diagnosed at an early stage obtain favorable effects when receiving conventional therapies, such as surgical resection, liver transplantation, or local ablation [2]. While for patients at advanced or metastatic stages, the effective treatment that prolongs HCC survival is limited to multiple tyrosine kinase inhibitors, including first- (e.g., sorafenib) [4] and second-line agents (e.g., regorafenib) [5]. Although clinical benefits have been reported using these drugs, the median survival interval is still less than 2 years. Therefore, more effective therapeutic approaches are urgently needed for advanced HCC patients.

In the past few years, immune checkpoint inhibitors (ICI) therapies, which reactivate the related regulatory signaling of T cells and revive the immune system of tumor patients to kill tumor cells, have remarkably extended the life expectancy in patients with distinct solid tumors [6, 7]. Owing to the favorable clinical efficacy, the Food and Drug Administration (FDA) has approved 4 immune checkpoint inhibition-based agents (i.e., ipilimumab, nivolumab, pembrolizumab and atezolimumab) for treatment of advanced stage cancers or metastases, such as melanoma and non-small cell lung cancer (NSCLC) [8]. The immune checkpoints directed against monoclonal antibodies from the above agents include cytotoxic T-lymphocyte protein 4 (CTLA-4), the programmed cell death protein 1 (PD-1), and its ligand, PD-L1 [9]. Nevertheless, only a minority of patients could obtain a durable treatment response to these regimens [10]. High PD-L1 expression is a frequently-used indicator to predict the efficacy of anti-PD-1 therapy [11–13]. Previous experimental evidence revealed that the presence of a high T cell infiltration, an interferon-gamma (IFN-γ) signature, checkpoint gene (e.g., PD-1 and PD-L1) expression, or a high tumor mutational load (TML) could favor a treatment response [14–16]. Conversely, several immune-suppressive factors, such as stromal cells and M2 macrophages infiltration, may lead to a reduction in the anti-tumor immune response, and resistance to ICI therapy [17]. In a phase I/II HCC clinical trial, remarkable responses were reported when patients were treated with nivolumab, a monoclonal antibody targeting PD-1 [18]. Unfortunately, there is less evidence relevant to the immunologic subtypes of HCC and how to make use of this information to achieve the best efficacy from immune checkpoint-based treatment.

The HCC microenvironment is a mixture of distinct cell types, including malignant hepatocytes, immune cells, endothelial cells, and stromal cells. A variety of analytic methods have been established to virtually extract molecular features from the tumor-immune microenvironment [19, 20]. By applying a non-negative matrix factorization (NMF) algorithm [21], we deconvoluted the gene expression profile of 719 HCC patients and dissected the signals related to the immune microenvironment, which allowed us to determine a potential immune subtype of HCC with specific immunologic features. The key traits of this subtype include infiltration of immune cells, increased enrichment of the IFN-γ signature, a T cell-inflamed signature and cytolytic activity, elevated expression of immune checkpoints, and most importantly, a favorable prognosis. The HCC immune subtype in our study may provide a novel strategy for evaluating survival and immunotherapy implications. Further in-depth investigations are warranted based on the HCC patients who received immunotherapy.

## RESULTS

### Identification of an immune class for HCC

**Figure 1:**
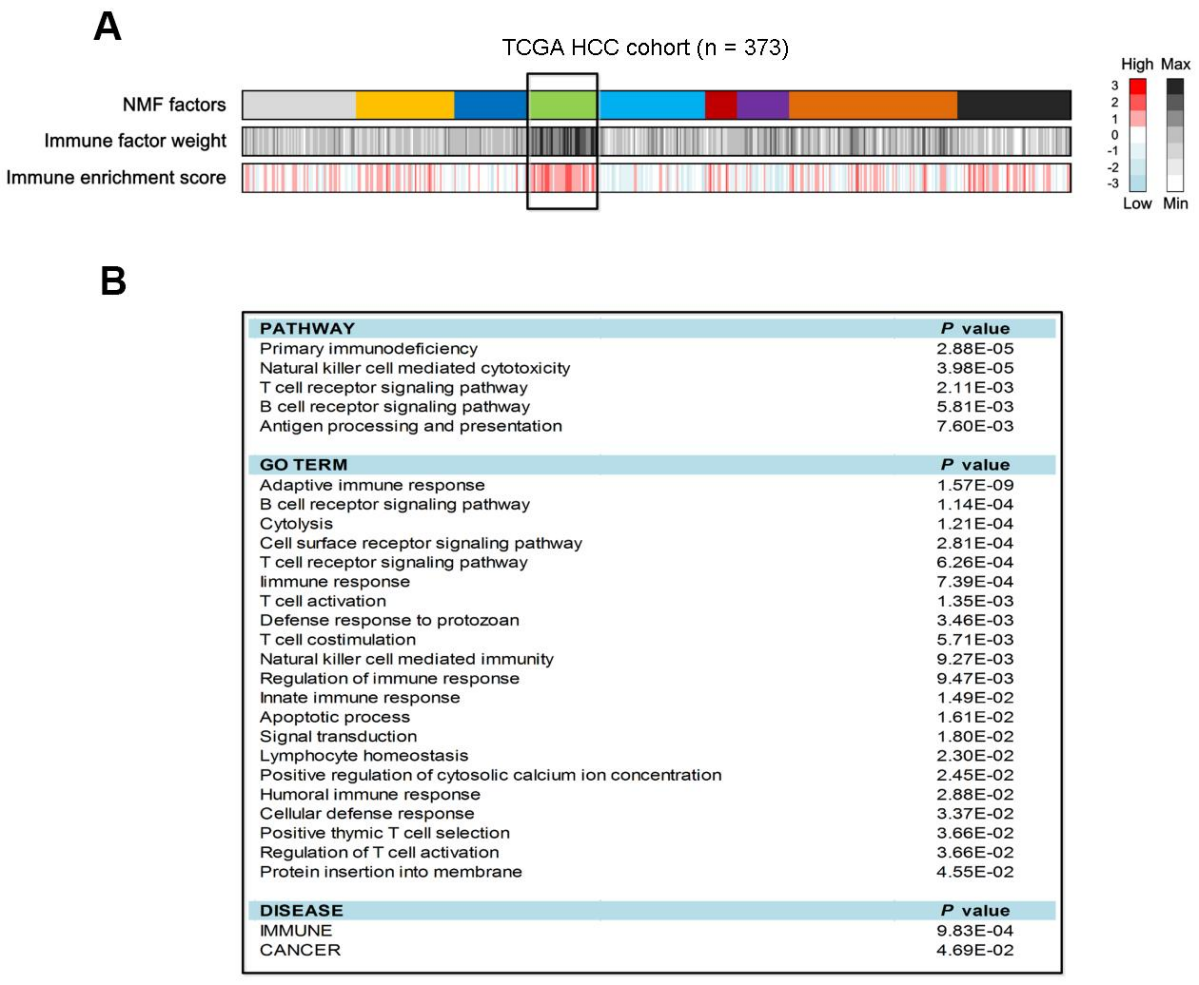
**Identification of HCC immune class and its pathway analysis in the TCGA cohort.** (**A**) The association of identified 9 HCC classes with immune enrichment score. (**B**) Pathway analysis of the top 100 genes contributed to immune class.

Coefficient of variation (CV) analysis showed that 8163 genes had CV values less than 0.1 in the TCGA cohort. Based on the gene expression profile of these genes, 9 classes were identified using NMF clustering analysis (Figure 1A). We found that one class harbored the highest immune enrichment score than others (Figure 1A), thus designated as the ‘immune class’. To verify the functionality of this immune class, we obtained the top 100 genes (Supplementary Table 1) that had the greatest contribution to this class to perform pathway annotation. Based on the results of pathway analysis, we observed that antigen processing and presentation, and signaling mediated by immune cells (e.g., T cells, B cells and NK cells) were significantly enriched (all *P* < 0.05; Figure 1B). Biology processes, such as innate and adaptive immune responses, T cell and B cell receptor signaling, T cell activation, and cytolysis were also observed (all *P* < 0.05; Figure 1B). Together, these findings further confirm that this immune class is immunogenic.

### Identification and validation of an immune HCC subtype

**Figure 2:**
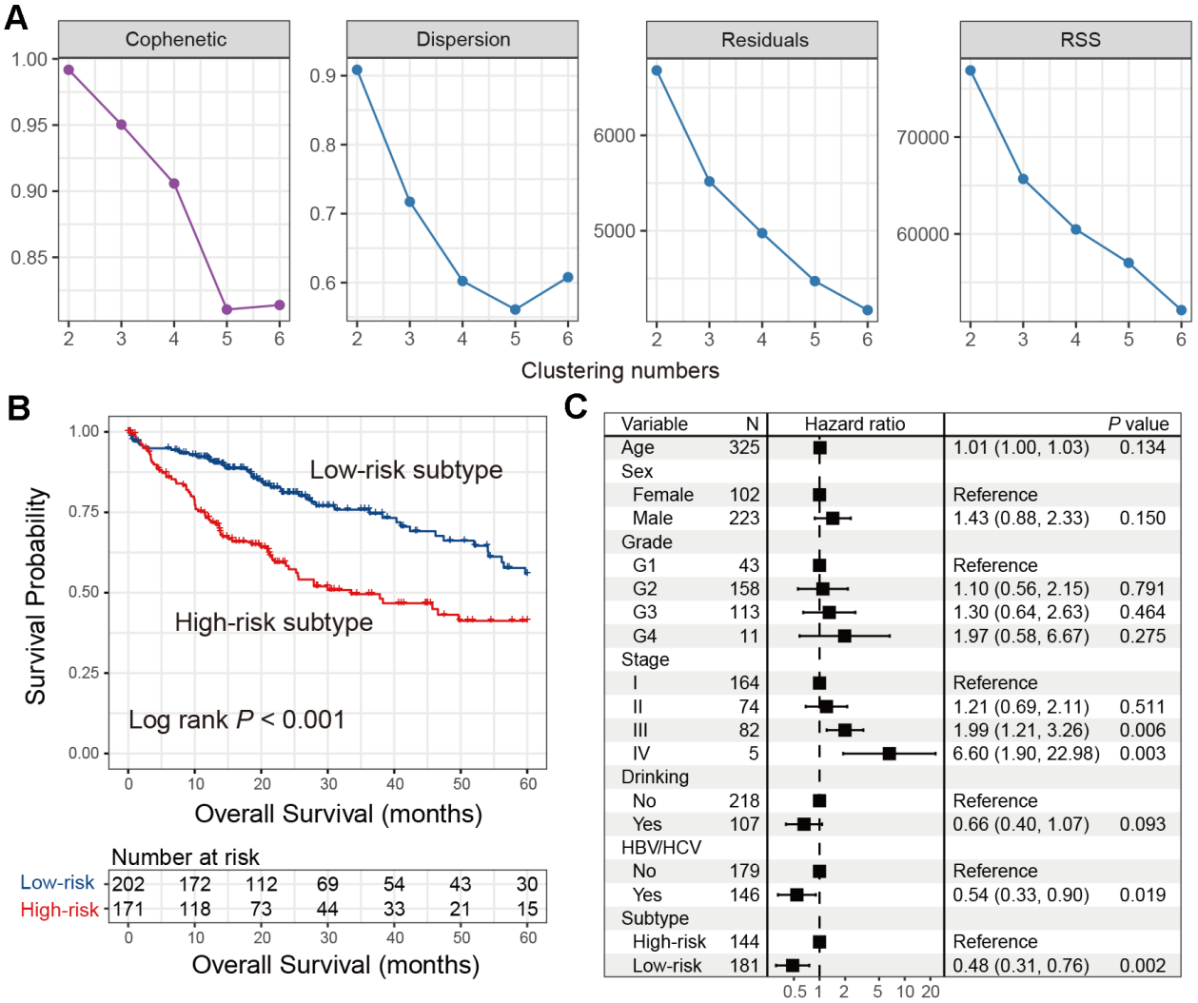
**Identification of the immune low-risk subtype of HCC in TCGA.** (**A**) Associations between NMF coefficients and clustering numbers. (**B**) Kaplan-Meier survival analysis of identified low-risk and high-risk subtypes. (**C**) Forest plot of multivariate Cox regression model with HCC clinical factors taken into account.

To obtain more accurate HCC subtypes, we performed NMF clustering based on the gene expression profile of the aforementioned top 100 genes of the immune class in the TCGA cohort. We separately evaluated the model parameters with clustering numbers set as 2-6. Cophenetic, dispersion, residuals, and RSS coefficients could obtain the maximum values when the cluster number was selected as 2 (Figure 2A) Consistently, heatmap analysis also exhibited the best clustering effect when the number was 2 (Supplementary Figure 1). We consider that two subtypes potentially exist in HCC patients.

Kaplan-Meier survival analysis showed that these two subtypes were statistically prognostically different (Log-rank test *P* < 0.001; Figure 2B). The subtype with better prognosis was designated as the ‘low-risk’ subtype (*n* = 202), and the subtype with poor survival was designated as the ‘high-risk’ subtype (*n* = 171). Multivariate Cox regression model with clinical characteristics and confounding factors (i.e., age, sex, grade, stage, drinking status, and HBV/HCV status) taken into account was still statistically significant (HR: 0.48, 95% CI: 0.31-0.76, *P* = 0.002; Figure 2C). Differential analyses results of the top 100 immune genes between low- and high-risk HCC subtypes in the TCGA cohort were shown in Supplementary Table 2.

**Figure 3:**
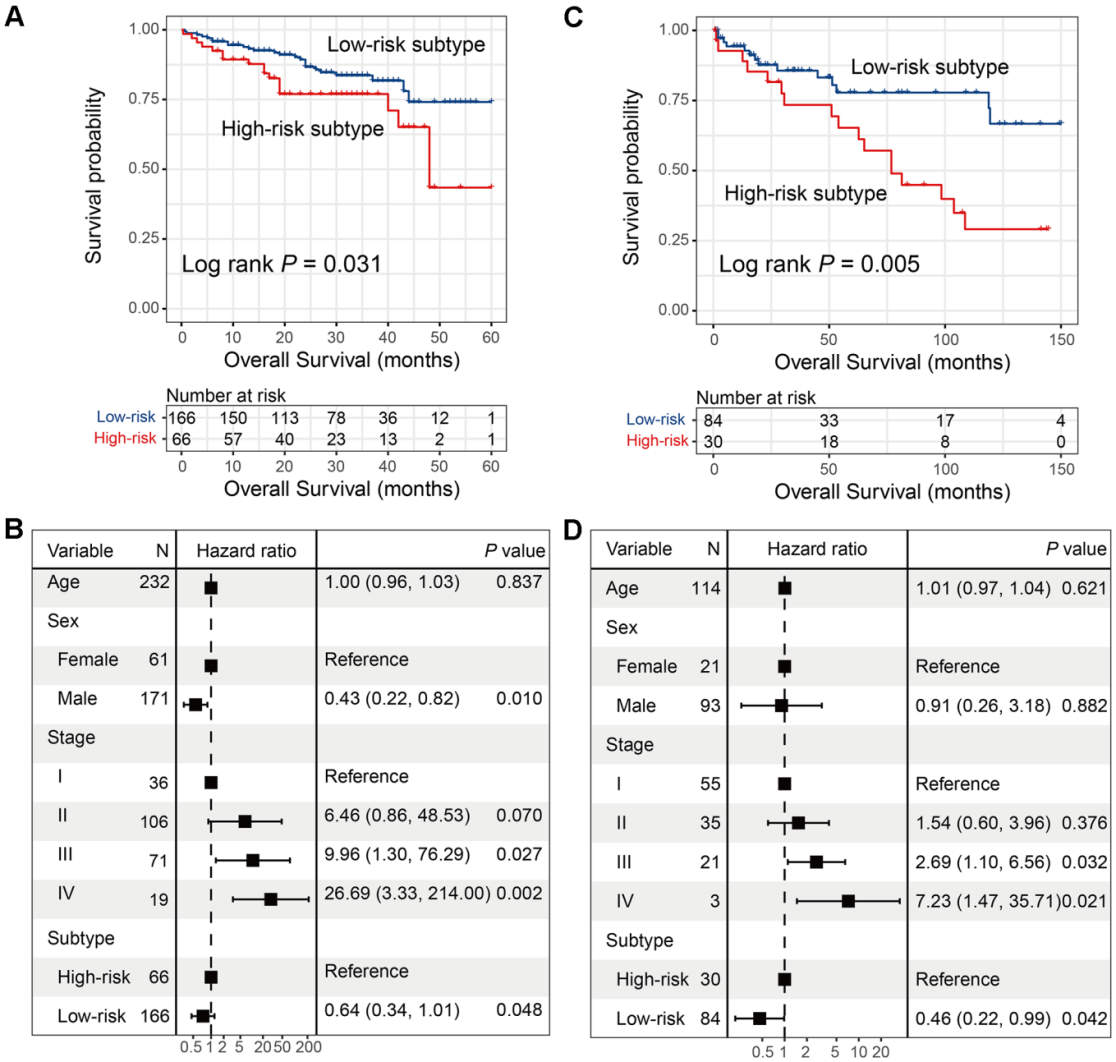
**Validation for the 2 HCC subtypes with additional 2 independent datasets.** (**A**, **B**) Univariate and multivariate survival analysis of 2 HCC subtypes in the ICGC cohort. (**C**, **D**) Univariate and multivariate survival analysis of 2 HCC subtypes in GSE76427.

Two independent HCC cohorts were utilized to validate the prognostic ability of the two subtypes identified from TCGA. Low- and high-risk subtypes were also observed via univariate analysis and the multivariate Cox regression model in ICGC cohort (Log-rank test *P* = 0.031; HR: 0.64, 95% CI: 0.34-1.01, *P* = 0.048; Figure 3A, 3B), as well as the GEO cohort (Log-rank test *P* = 0.005; HR: 0.46, 95% CI: 0.22-0.99, *P* = 0.042; Figure 3C, 3D).

### Patients from the low-risk HCC subtype harbored immune-activated microenvironment

To elucidate the association of low-risk subtype with better prognosis, we explored the vital factors in the microenvironment in relation to the low-risk subtype.

**Figure 4:**
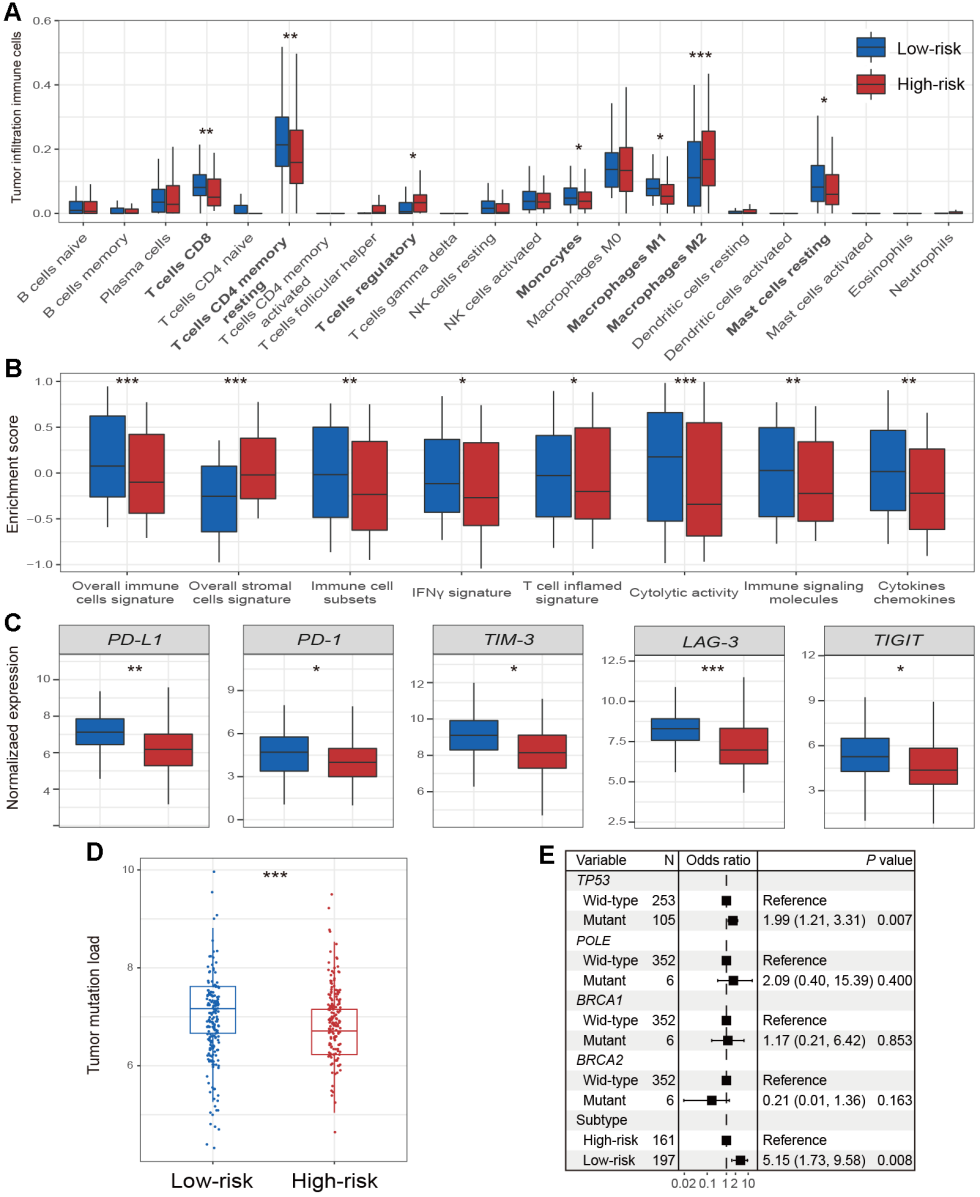
**Immune microenvironment and genomic features in relation to 2 HCC subtypes.** Distinct enrichment of (**A**) infiltration immune cells, (**B**) immune-related signatures, and (**C**) immune checkpoints in the 2 HCC subtypes. (**D**) The association of 2 identified subtypes with TML. (**E**) Forest plot representation of the association between the 2 identified subtypes and TML. * *P* < 0.05, ** *P* < 0.01, *** *P* < 0.001.

For the infiltration of immune cells, we found that the low-risk subtype had significantly higher CD8 T cell infiltration than the high-risk subtype (*P* < 0.01; Figure 4A). In addition, resting CD4 memory T cells, monocytes, and resting mast cells were also significantly enriched in the low-risk subtype (all *P* < 0.05; Figure 4A). Enrichment of the regulatory T cells, which exhibit the immune suppression, was markedly decreased in the low-risk subtype (*P* < 0.05; Figure 4A). Interestingly, the low-risk subtype had significantly elevated infiltration of M1 macrophages (*P* < 0.05; Figure 4A), which is one subtype of macrophages that promotes the inflammation. M2 macrophages, which are associated with tumor growth and immune inhibition, were significantly enriched in the high-risk subtype, compared to the low-risk subtype (*P* < 0.001; Figure 4A).

Patients of low-risk subtype had significantly higher enrichment of total immune cells, and immune cell subsets (i.e., T cells, B cells, and NK cells) (all *P* < 0.01; Figure 4B). The enrichment of immune-suppressive stromal cells was significantly decreased in the low-risk subtype (*P* < 0.001; Figure 4B). In the low-risk subtype, we also found markedly increased enrichment of the IFN-γ signature and the T cell-inflamed gene signature (both *P* < 0.05; Figure 4B), which were recently reported to be correlated with ICI efficacy [22]. In addition, enhanced cytolytic activity, and elevated enrichment of cytokines and chemokines were all observed in patients from the low-risk subtype (all *P* < 0.01; Figure 4B).

We found that expression of *PD-L1* and *PD-1* was significantly upregulated in low-risk subtype patients (both *P* < 0.05; Figure 4C). Other checkpoints, including *TIM-3*, *LAG-3*, and *TIGIT* also obtained similar results (all *P* < 0.05; Figure 4C); however, no differences were detected in *CTLA-4* and *IDO1* expression between low- and high-risk subtypes (both *P* > 0.05; Supplementary Figure 2).

We also observed that the low-risk HCC subtype harbored a significantly higher TML than high-risk subtype (*P* < 0.001; Figure 4D). Multivariate Logistic regression model with mutations of DNA repair genes (i.e., *TP53*, *POLE*, *BRCA1*, and *BRCA2*) taken into consideration was still significant (OR: 5.15, 95% CI: 1.73-9.58, *P* = 0.008; Figure 4E).

### Pathways significantly enriched in the low-risk HCC subtype

**Figure 5:**
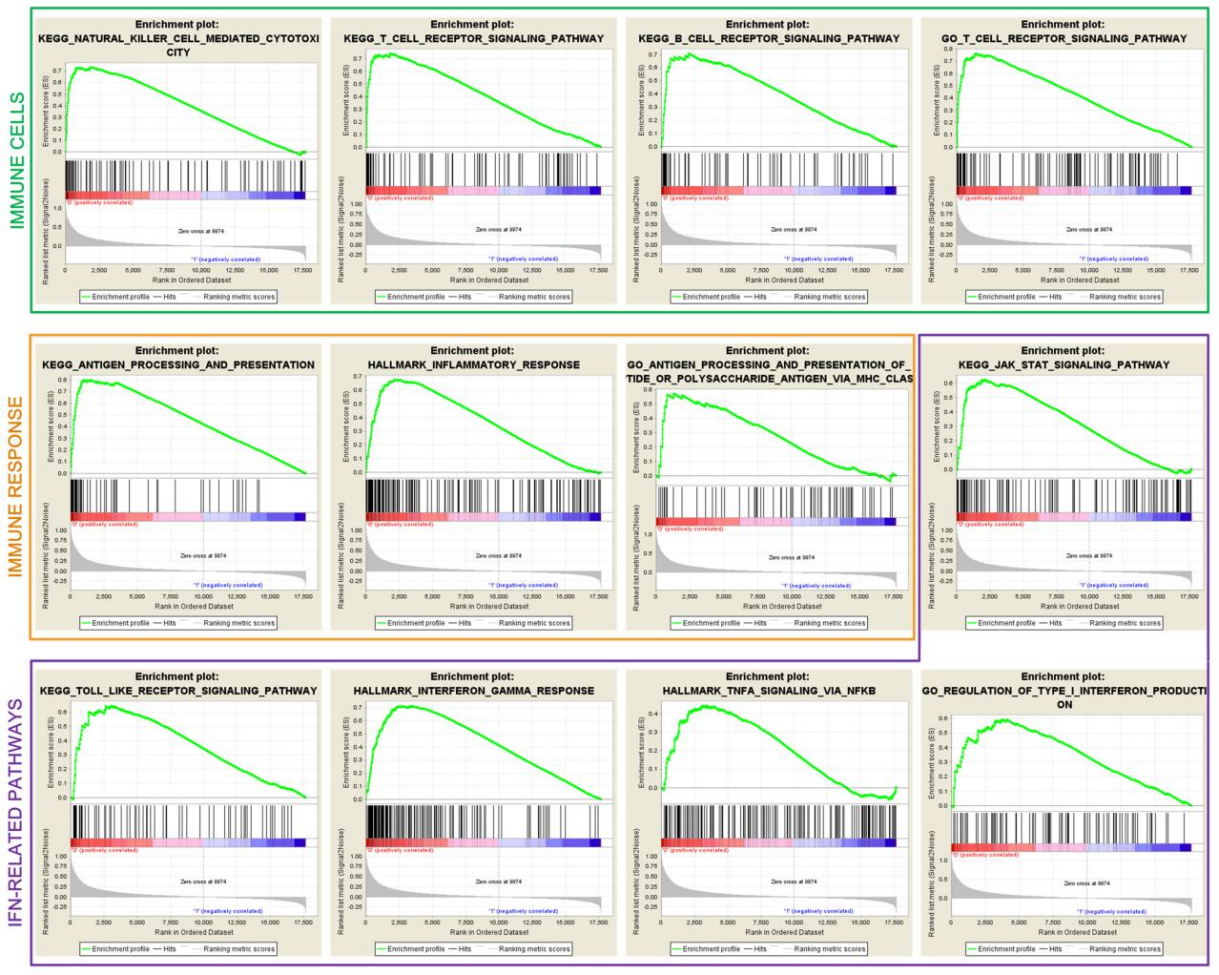
Significantly enriched immune cells, immune response, and IFN-related pathways in the low-risk subtype.

GSEA analysis between the two subtypes showed that immune cell-related pathways, such as NK cell-mediated cytotoxicity, and T and B cell receptor signaling pathways were markedly enriched in the low-risk subtype (all FDR < 0.05; Figure 5). Immune response pathways including antigen processing and presentation, and the inflammatory response were also enriched (all FDR < 0.05; Figure 5). Patients of low-risk subtype harbored the enrichment of IFN-γ-related pathways, which are associated with anti-tumor immunity and immunotherapy efficacy (all FDR < 0.05; Figure 5).

### SMGs of the low-risk HCC subtype

**Figure 6:**
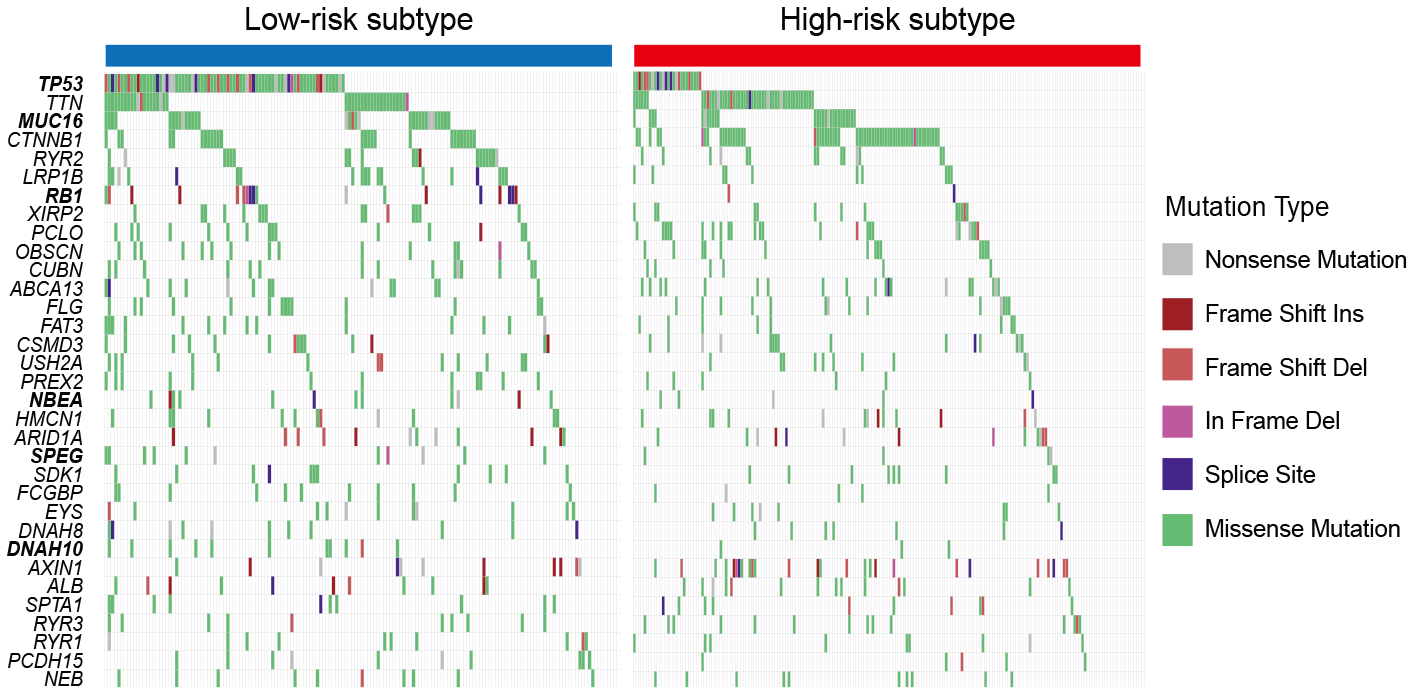
**Mutation rates of SMGs stratified with the 2 HCC subclasses.** Genes with bold and italic font were observed to be significantly differentially mutated in the 2 HCC subtypes. * *P* < 0.05, ** *P* < 0.01, *** *P* < 0.001.

A total of 33 significantly mutated genes (SMGs) were identified using the MutSigCV algorithm. Differential analyses of the SMGs mutation rates between the two subtypes showed that *TP53*, *MUC16*, *RB1*, *NBEA*, *SPEG*, and *DNAH10* mutations were significantly enriched in the patients of low-risk subtype (Fisher exact test, all *P* < 0.05; Figure 6). Among them, *TP53* mutations were previously reported to be associated with the ICI response in lung adenocarcinoma (LUAD), and *MUC16* mutations harbored potential immunotherapy implications for gastric cancer (GC) patients.

## DISCUSSION

By using virtual dissection analytic methods, we deconvoluted the gene expression profile from mixed HCC tissues, and thus determined an undiscovered immune pattern and a moderate immunology subtype of HCC, herein designated the low-risk subtype. The low-risk subtype harbored favorable survival outcomes, a better immune microenvironment, and genomic features compared to the high-risk subtype. The identified low-risk subtype may be a promising indicator for prognosis prediction and clinical immunotherapy of HCC patients.

The prognoses of patients with melanoma and NSCLC have dramatically changed owing to the approval of ICI agents by the FDA. Long-range clinical benefits and durable remissions induced by these agents have been observed in a subset of patients with metastatic or advanced stage cancer [10, 23]. Taking into consideration that the directed targets of these drugs are immune cells instead of tumor cells, the effective responses could be detected in multiple cancers, such as colorectal [13, 16] and bladder cancer [24]. In the phase II clinical trial that involved 214 HCC patients who received nivolumab, the objective response rate and median survival interval were 16% and 14 months, respectively [18]. In this trial, patients with clinical responses were not shown to be associated with high PD-L1 expression [18]. Therefore, the determination of more reliable indicators to select the appropriate sub-population for receiving ICI therapy is urgently needed for HCC. Patients negative for PD-L1 sometimes exhibit durable benefits. This observation further indicates the instability of PD-L1 expression as a biomarker; novel moderate biomarkers are needed to be investigated.

In this study, a novel immune subtype, herein designated the low-risk subtype of HCC, was identified and crucial insights into the immunologic features of this subtype were provided. Patients with the low-risk subtype, whose molecular traits, including an abundance of infiltration immune cells, enhanced enrichment of immune-related signatures, high expression of immune checkpoints, and an elevated TML, highly resemble the tumors that are responsive to ICI agents [14–16]. IFN-γ and the T cell-inflamed gene signature, which were previously reported to predict the efficacy of pembrolizumab [25], were demonstrated in HCC patients with the immune subtype. This finding reinforces the inference that ICI responses may appear in tumors with a pre-existing IFN-mediated immune signaling. Interestingly, the presence of high TML was observed in the low-risk immune subtype, indicating that, unlike melanoma [16, 26] and NSCLC [26], distinct potential mechanisms may actuate HCC immune responses. Prostate, ovarian, and pancreatic tumors with modest TML also exhibit a similar lack of association [27]. In these situations, the immunogenicity of tumors may be influenced by neoantigen quality, rather than quantity [26]. In addition, several mutation-independent signals, for example, HCC-related antigens expression, may induce a vital effect on the anti-tumor immune response [8].

Differential analysis of SMG mutations showed that six genes exhibited higher mutation rates in the low-risk subtype than high-risk subtype. Among these six SMGs, *TP53* mutations were reported to be associated with high expression of immune checkpoints, an active IFNγ signature, and effector T cell signature, and favorable anti-PD-1 efficacy in LUAD [28]. A recent study revealed that *MUC16* mutations are significantly correlated with a higher TML and better survival outcomes in patients with GC [29]. We demonstrated that patients with the low-risk subtype harbored higher mutation rates of the 2 SMGs further verify the predictive roles of this subtype in immune checkpoint-based therapy.

By using the Nearest Template Prediction (NTP) algorithm with 1950 representative meta-genes, Hoshida et al. identified three HCC subtypes (i.e., S1, S2, and S3) that were correlated with distinct biological processes [30]. We also employed the NTP method with the same meta-genes to determine Hoshida et al. subtypes in the three HCC datasets included in our study, and compared them with the low- and high-risk subtypes we identified. In the TCGA and ICGC cohorts, we showed that the low-risk subtype harbored a significantly decreased proportion of S1subclass (Supplementary Figure 2A, 2B), which was characterized by WNT-TGFβ pathway activation. Recent studies have demonstrated the WNT-TGFβ signal functions in immune suppression [31, 32]. The lower proportion of S1 further verified the immunotherapy implications of patients from the low- risk subtype; however, no significant distribution differences were observed between the low/high-risk subtypes and the S2/S3 subclasses. The distribution differences of the 3 subclasses in low- vs. high-risk subtypes were not statistically significant in GSE76427 (Supplementary Figure 2C); this may be owing to the smaller sample size of this cohort.

Taking into consideration the important effects of ethnicity on precision medicine, we therefore compared the race distribution between HCC low- and high-risk subtypes. We observed that there is no significantly distinct race distribution in low- vs. high-risk subtypes (Fisher exact test *P* = 0.271; Supplementary Figure 3).

Meanwhile, several inadequacies existed in our study. First, the gene expression data were acquired from distinct sequencing or microarray platforms, which may introduce result biases in the analysis. Second, somatic mutation data used in this work were only from the TCGA project; no additional HCC datasets contained mutation profiles were available.

Our study discovered a novel immune subtype of HCC patients that was associated with favorable survival outcomes and a better immune microenvironment, who may represent the appropriate sub-populations to receive ICI agents. In-depth explorations of this immune subtype in larger immunotherapy cohorts are needed to validate its potential utility as a predictive indicator of response to ICI therapies.

## MATERIALS AND METHODS

### Gene expression profile, somatic mutation data, and clinical information of used HCC patients

A total of 373 HCC patients with mRNA expression profile and follow-up information in the Cancer Genome Atlas (TCGA) were obtained from Genomic Data Commons (https://gdc.cancer.gov/). Among them, 325 patients had complete clinical characteristics (i.e., age, sex, grade, stage, drinking status, and HBV/HCV status). From International Cancer Genome Consortium (ICGC) [LIRI-JP cohort] and Gene Expression Omnibus (GEO) [accession number: GSE76427], we respectively acquired 232 and 114 patients with gene expression and clinical data for further validation (Supplementary Table 3). All gene expression data were normalized for subsequent analyses. For genes with multiple probe sets, the mean gene expression was utilized as the expression level. Somatic mutation data of 364 patients with mRNA expression were obtained from the TCGA cohort. In this study, non-synonymous mutational types, including missense mutation, nonsense mutation, frame shift del/ins, in frame del/ins, and splice site mutation were included to perform related analyses.

### NMF clustering analysis

Clustering analyses based on mRNA expression profile were conducted with non-negative matrix factorization (NMF) method embedded in the R NMF package [21]. A binary matrix A representing gene expression levels (rows) across HCC patients (columns) was generated. Then, expression matrix A was divided into two non-negative matrices W and H (i.e., A≈WH). Distinct classes or subtypes were identified with a clustering approach based on Matrix H. Optimal clustering number was determined according to the values of cophenetic, dispersion, residuals and RSS coefficient.

### Identification of immune expression patterns and potential immune subtypes

Tumor, stromal and immune cell gene expression data from the TCGA cohort were virtually microdissected using abovementioned NMF algorithm. In this study, genes with a CV less than 0.1 were selected to reduce the biases of results [33]. We selected the number of clustering factor as 9, as it could effectively divide the expression data in the TCGA cohort, and thus exhibit a high cophenetic coefficient [34].

Then we took the following steps to identify an immune class as previously reported by Sia et al. [8]. Firstly, an immune enrichment score gene signature [20], which represents the proportion of infiltration immune cells in tumor tissue, was utilized to determine the potential immune relevant class (or expression pattern). By integrating all 9 NMF-identified clusters with the immune enrichment score gene signature, we observed the NMF cluster with the highest immune enrichment score, and named this as the immune class. Then, we curated the top 100 genes based on their contributions to the immune class, and these 100 genes were annotated with the DAVID tool (https://david.ncifcrf.gov/) to further verify their immune functionalities. Finally, the top 100 genes were utilized to perform unsupervised NMF clustering analysis to divide the TCGA HCC patients into distinct subtypes.

### Infiltration immune cells, immune-related signatures, and immune checkpoint genes in microenvironment

Proportion of tumor infiltration immune cells was estimated with CIBERSORT algorithm, which is an analytical tool developed by Newman et al. to provide the calculated abundances of 22 immune cell types in the mixed tumor tissue, using the LM22 signature based on gene expression data [35].

Recently reported vital immune-related signatures that represented distinct immunology and cellular statuses were collected as follows: 1) overall immune cells and stromal cells signature, which indicates the infiltration proportion of total immune cells and stromal cells in mixed tumor tissue [20]; 2) immune cell subsets, which represents the enrichment of T cells, B cells and NK cells [36]; 3) IFNγ signature, which is a signal correlated with anti-tumor immune response and ICI efficacy [25]; 4) T cell-inflamed signature, which is a signature with high expression of dendritic cell and CD8^+^ T cell-associated genes, and this signature was also reported to have a positive link with immunotherapy response [25]; 6) cytolytic activity [37]; 7) immune signaling molecules [36]; 8) cytokines and chemokines [36].

Immune checkpoints in current ICI therapy mainly contain PD-L1, PD-1 and CTLA-4 [38, 39]. Other checkpoints, for instance, LAG-3, TIM-3, TIGIT and IDO1, which are undergoing clinical trials, play vital roles in checkpoint blockade treatment [40–43].

Besides the microenvironment-based immune factors in relation to distinct subtypes, we also evaluated the association of potential subtypes with TML with univariate analysis and multivariate regression model.

### Gene set enrichment analysis

Single sample gene set enrichment analysis (ssGSEA) method embedded in R package GSVA (version 1.32.0) was utilized to calculate the enrichment scores of above immune signatures for each sample [44]. We used gene set enrichment analysis (GSEA) from fgsea package (version 1.10.0, https://bioconductor.org/packages/fgsea/) to explore the dysregulated pathways in distinct subgroups. Annotated pathways in Molecular Signature Database (MSigDB, version 3.0) [45] were utilized as the background signals.

### Significantly mutated genes

Significantly mutated genes (SMGs) were determined by applying MutSigCV method [46]. The significant enrichment of non-silent somatic variants of a specific gene was calculated by MutSigCV via addressing mutational context specific background mutation rates. The following criteria were needed to authenticate SMGs: statistically significant (i.e., q value less than 0.1), expressed in TCGA HCC data [47] and encyclopedia of cell lines [48], and mutation rate greater than 5%.

### Statistical analyses

R software (version 4.0.3) and its packages were utilized to perform statistical analyses and produce relevant figures. Kaplan-Meier survival curves were generated with R survival (version 2.44-1.1) and Survminer (version 0.4.5) packages, and the Log-rank test was used to compare the difference between survival curves. In addition to univariate analyses, multivariate Logistic and Cox regression models with confounding factors taken into consideration were performed using Forestmodel package (version 0.5.0) to control the false positive. Associations of distinct subtypes with continuous and categorical variables were calculated with Wilcoxon rank-sum test and Fisher exact test, respectively. In this study, *P* values less than 0.05 were considered to be statistically significant, unless special instructions.

## Supplementary Material

Supplementary Figures

Supplementary Table 1

Supplementary Table 2

Supplementary Table 3
