# Supplementary material for: Identification of an immune subtype predicting survival risk and immune activity in hepatocellular carcinoma
Source: Aging (Albany NY). 2021 May 3;17(11):2859–74. doi: 10.18632/aging.202953 (PMC12705184; doi:10.18632/aging.202953)
Supplement: Supplementary Figures [file aging-17-11-202953-s001.pdf]

SUPPLEMENTARY FIGURES

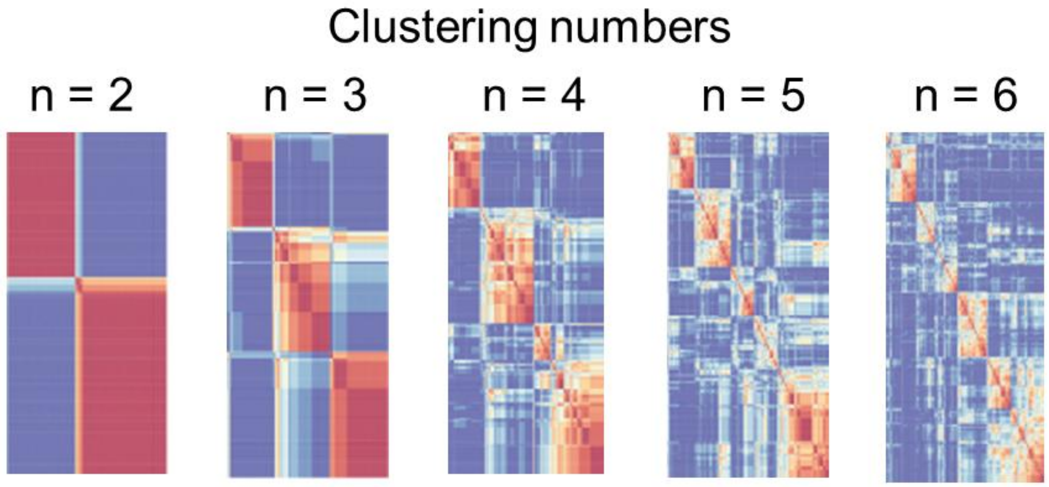

Supplementary Figure 1. Heatmap presentation of clustering results of HCC patients.

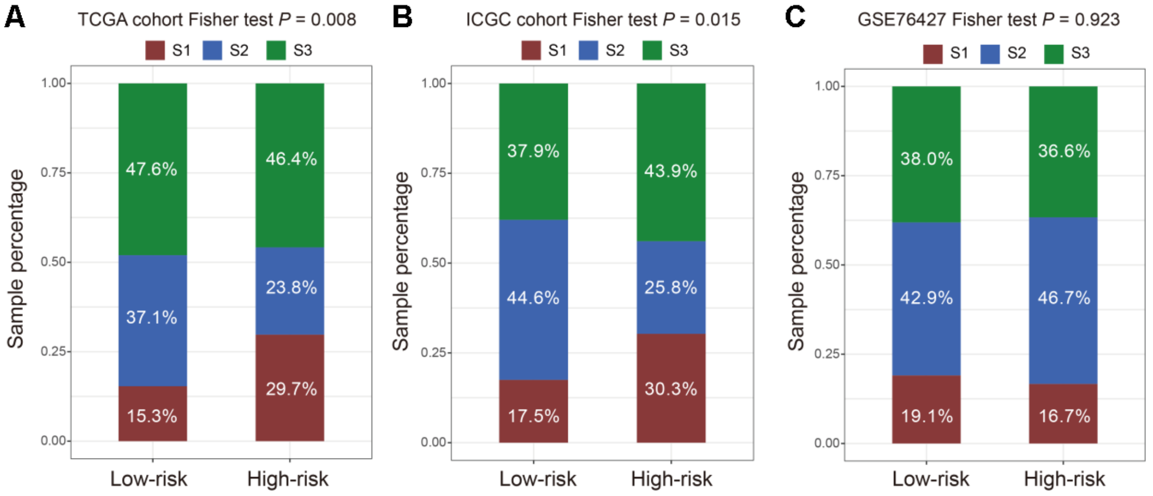

Supplementary Figure 2. Distribution of Hoshida et al. three HCC subclasses in low- vs. high-risk subtypes in (**A**) TCGA cohort, (**B**) ICGC cohort, and (C) GSE76427 cohort.

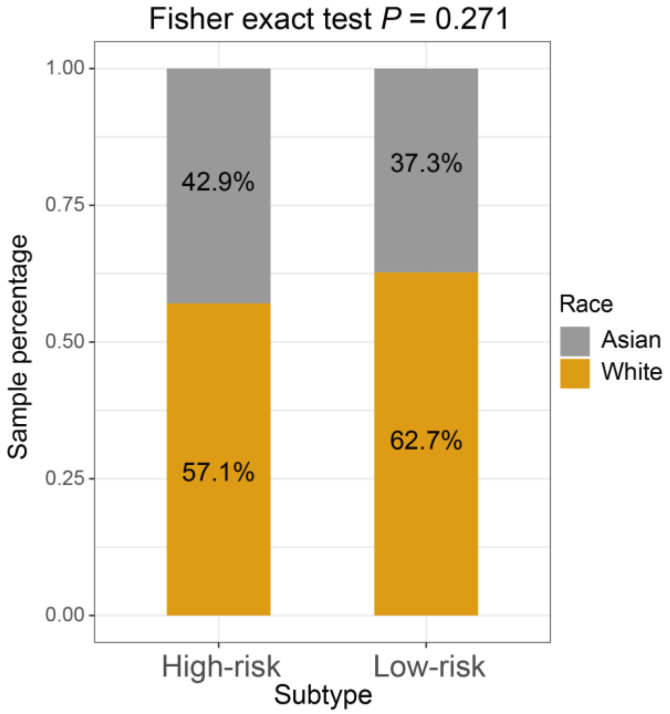

Supplementary Figure 3. Distinct race distribution in HCC low- and high-risk subtypes in the TCGA cohort.
