# Supplementary material for: Identification of an immune subtype predicting survival risk and immune activity in hepatocellular carcinoma
Source: Aging (Albany NY). 2021 May 3;17(11):2859–74. doi: 10.18632/aging.202953 (PMC12705184; doi:10.18632/aging.202953)
Supplement: Supplementary Table 3 [file aging-17-11-202953-s004.pdf]

SUPPLEMENTARY TABLE

Supplementary Table 3. Information of three datasets included in this study.

Datasets	Datasets	Datasets
TCGA	Illumina RNAseq HTSeq	373
ICGC	Illumina RNAseq HTSeq	232
GSE76427	Illumina HumanHT-12 V4.0 expression beadchip	114
Total		719
